# Assessment of Oxidative Stress and Associated Biomarkers in Wild Avian Species

**DOI:** 10.3390/ani15091203

**Published:** 2025-04-23

**Authors:** Siniša Faraguna, Suzana Milinković Tur, Sandra Sobočanec, Marija Pinterić, Maja Belić

**Affiliations:** 1 Department of Pathophysiology, Faculty of Veterinary Medicine, University of Zagreb, 10000 Zagreb, Croatia; sfaraguna@vef.unizg.hr; 2 Department of Physiology and Radiobiology, Faculty of Veterinary Medicine, University of Zagreb, 10000 Zagreb, Croatia; tur@vef.unizg.hr; 3 Laboratory for Metabolism and Aging, Division of Molecular Medicine, Ruđer Bošković Institute, 10000 Zagreb, Croatia; ssoboc@irb.hr (S.S.); marija.pinteric@irb.hr (M.P.)

**Keywords:** antioxidants, wild birds, biomarkers, oxidative damage, oxidative stress

## Abstract

Reactive oxygen species (ROS) are natural by-products of cellular metabolism and are also formed in response to environmental stressors such as pollution, extreme temperatures, and ultraviolet radiation exposure. Physiological factors such as intense activity, growth, reproduction, nutrient deficiency, captivity, and disease also contribute to ROS production. While ROS, including free radicals, play a key role in cell physiology, including immune defense, their excessive accumulation can damage cellular components and cause oxidative stress when antioxidant defenses are overwhelmed. To regulate ROS levels, wild birds rely on enzymatic (e.g., catalase, superoxide dismutase, glutathione peroxidase) and non-enzymatic antioxidants (e.g., vitamins C and E, carotenoids). Oxidative stress affects important aspects of wild bird biology, including health, reproduction, and survival, and is closely linked to overall fitness. It is also linked to physiological challenges such as migration and the progression of various diseases affecting wild bird populations. The study of oxidative stress in wild birds requires the use of appropriate biomarkers to assess its role in disease development. A deeper understanding of the balance between ROS production and antioxidant defenses is essential to determine how wild birds cope with environmental and physiological challenges. In this review, we summarize the mechanisms of oxidative stress in wild birds and the role of antioxidants in maintaining health and promoting longevity in wild bird populations.

## Simple Summary

Wild birds face many ecological and physiological challenges, such as pollution, intense activity, migration, and disease, which can lead to the production of harmful molecules called reactive oxygen species. While these molecules are naturally produced in the body and can support immune function, too many of them can damage cells and cause a condition known as oxidative stress. Birds have natural defenses, known as antioxidants, that can protect them from this damage. These include certain enzymes and nutrients such as vitamins and pigments. If the balance between harmful molecules and protective antioxidants is disrupted, this can have negative effects on the health, reproduction, and survival of wild birds. By understanding how this balance works, scientists can learn how birds respond to stress and what affects their well-being. This review explains how oxidative stress occurs, how it affects bird populations, and how antioxidants help birds stay healthy. These findings are valuable for protecting wild birds and supporting conservation efforts.

## 1. Introduction

**Figure 1:**
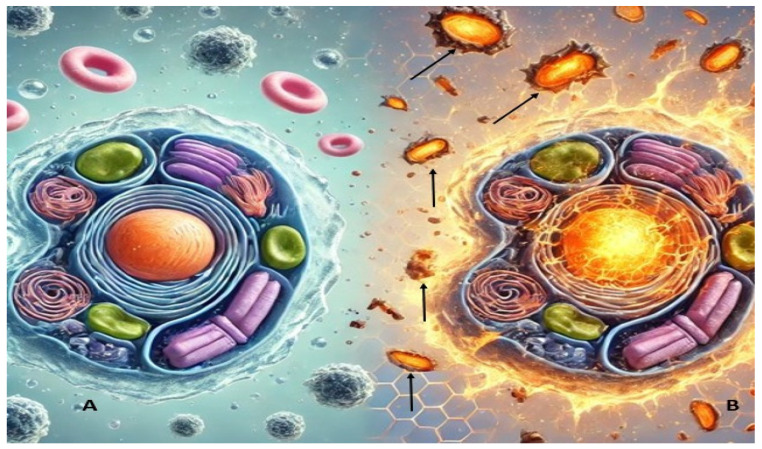
(**A**) A healthy animal cell; (**B**) effect of free radicals on an animal cell. Black arrows show free radicals attacking the cell.

The allocation of energy between reproduction and self-preservation is an important trade-off in the history of life. Therefore, the forces that drive the constant renewal of organic tissues have been examined to understand their role in shaping life. When an organism encounters a harmful substance, it initiates an energy-intensive process to counter the harmful effects or enable these effects to be tolerated. Over time, natural selection has favored organisms that can effectively detoxify and potentially utilize harmful substances [1]. Oxygen is one such substance. Since aerobic energy production began about 3 billion years ago, organisms have risen to the challenge of respiration [2]. This process of electron shifting inevitably produces molecules with unpaired electrons, known as reactive species [3]. These include reactive oxygen species (ROS), reactive nitrogen species (RNS), and others that endanger biological structures (Figure 1) [4,5]. Over billions of years, organisms have evolved antioxidant mechanisms to mitigate the harmful effects of these reactive species [6]. These mechanisms, such as the protection of vital structures, the production of scavengers of reactive species, and the repair of damaged biomolecules, have been refined over generations [7]. Notably, reactive species also act as weapons against pathogens [8] and as signaling molecules that can provide general stress signals within cells [9].

Given the versatility of reactive species, oxidative stress (OS) is defined as an imbalance between pro-oxidants and antioxidants that favors pro-oxidants and leads to impaired redox signaling and control, and/or molecular damage [10,11,12,13,14]. In addition to this biochemical definition, Costantini [15] has recently suggested that oxidative stress can be defined biologically as any alteration of one of the molecular components of the redox system that affects organismal fitness. Indeed, the intensity and duration of oxidative stress can influence the fate of the cell, ranging from an adaptive response to apoptosis or necrosis [16]. An adaptive cell response involves mechanisms and changes that a cell activates to protect itself when exposed to ROS. These mechanisms include upregulation of antioxidants, repair of damaged DNA, proteins, or lipids, or autophagy to allow the cell to survive [16]. The study of how this perturbation of redox homeostasis affects fitness by influencing sexual selection, reproduction, ageing, and survival is referred to as the ecology of oxidative stress [17]. The costs of reproduction are a central theme in evolutionary ecology. It has been hypothesized that oxidative stress may serve as a key factor contributing to these costs, this explaining the negative correlation between fecundity and longevity [18].

Studies on oxidative balance in urban and rural wild birds have proliferated dramatically in the 2010s and 2020s, but the most recent meta-analysis including urban studies was published over a decade ago by Isaksson [19]. To date, we have gleaned much information for various purposes from studies of domesticated mammals, but given the high metabolic rates and unusual resilience of many birds to oxidative damage [20,21], it is valuable to consider their patterns of antioxidant accumulation and activity, the sources of these, and their mechanisms for coping with oxidative stress [22].

In this review, we summarize the mechanisms of oxidative stress in wild birds and examine the role of antioxidants in maintaining and improving the health and longevity of wild bird populations.

## 2. Free Radicals and Oxidative Stress

In animals, cells continuously generate ROS, including free radicals, which play a key role in cell physiology and pathophysiology, such as in immune responses to destroy invading microorganisms [10]. Due to their high chemical reactivity, free radicals damage cells by attacking cellular components and organelles, especially proteins, lipids, carbohydrates, and DNA. Whether oxidative stress leads to cellular damage of biomolecules depends on the cellular balance of pro-oxidants and antioxidants, which can be considered as an equilibrium system in terms of homeostasis [23]. Free radicals are also involved in the induction or inhibition of various signaling pathways, the expression of specific genes, the induction or inhibition of cell proliferation, and the cell death process [24,25].

Free radicals can come from the outside (e.g., ionizing radiation, which can lead to the formation of free radicals through the radiolysis of water) as well as from internal processes, e.g., through enzyme systems of catalysis in the mitochondrial electron transport system (an example of random formation) [14,26,27]. The mitochondria are the primary site of free radical production in the cell, with most free radicals generated under physiological conditions by electron transport at the inner mitochondrial membrane during the conversion of oxygen to water as part of the electron transport chain during respiration [28]. In addition to the mitochondria, electron transport also takes place at the membranes of the endoplasmic reticulum and at the nuclear membrane, which leads to the formation of free radicals [29].

Biochemically, the most important free radicals are ROS and RNS, which are constantly produced in vivo during physiological metabolism in tissues [30,31]. ROS can be divided into two groups: radicals and non-radicals. Radicals are chemical particles that have at least one unpaired electron in their outer shells around the atomic nucleus and can exist independently as such. The oxygen molecule is also a radical, and since it contains two unpaired electrons, it is called a biradical. Other radicals include superoxide, hydroxyl, and peroxyl radicals. Non-radicals, which are not free radicals, can easily trigger reactions with free radicals in organisms. These non-radicals include hydrogen peroxide, hypobromous acid, hypochlorous acid, ozone, and singlet oxygen [32,33,34].

In addition to ROS, RNS also play an important role. RNS include radicals such as nitric oxide (NO) and nitrogen dioxide (NO_2_) as well as non-radicals such as nitric acid, nitrosyl cation, nitroxyl anion, nitrile cation, peroxynitrite, peroxynitrous acid, and alkyl peroxynitrates [33,35].

### Free Radicals and Oxidative Stress

Oxidative stress is thought to be responsible for the reduced life expectancy of wild birds. In particular, it is widely believed that cell proliferation is impaired by oxidative stress, leading to telomere shortening [36], and it has long been hypothesized that this is associated with a higher metabolic rate (i.e., life rate). The mechanism by which the production of reactive species depends on metabolic state remains controversial, as increased mitochondrial energy production can lower ROS levels [37]. However, higher growth rates have been associated with both increased and decreased antioxidant defenses and oxidative damage [38]. Oxidative damage to macromolecules has also been shown to increase with ageing, suggesting that the rate of senescence may be influenced by the balance between oxidative stress and membrane vulnerability [39].

## 3. The Influence of Environmental Stressors on Oxidative Stress in Wild Birds

**Figure 2:**
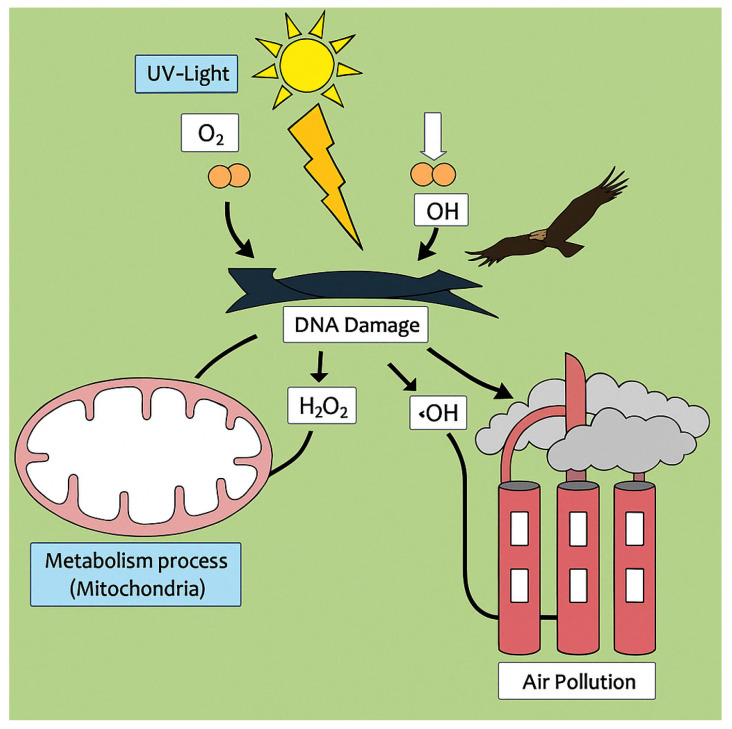
Formation of reactive oxygen species formation via UV light, metabolism processes, and air pollution, leading to cell damage (adapted from Manisha et al., 2017 [105]).

In the wild, animals are constantly exposed to a variety of stress factors (Figure 2). Ideal temperature, humidity and other environmental conditions are important for effective protection against the formation of free radicals. Various stressors (such as captivity, handling, pollution) are associated with an overproduction of free radicals and cause oxidative stress, i.e., a disturbance of the pro-oxidant–antioxidant balance leading to potential damage of macromolecules [15,40]. Several molecular and physiological parameters are also affected by the urban environment, including altered gene expression, endocrine changes, increased oxidative stress, and accelerated telomere attrition [41,42]. Comparative studies have shown that large-brained bird species perform better in terms of learning, cognition, innovation, and behavioral coping with environmental stressors, demonstrating enhanced survival and longevity in altered or novel environments [43,44,45,46]. Environmental stressors can be divided into three main categories. Among the most important of these is nutritional stress, including a deficiency of vitamin E, Se, Zn, or Mn, hypervitaminosis A, and the presence of various toxins (e.g., heavy metal poisoning) [15,47]. Urbanization can drastically change the availability of food for wild birds. Anthropogenic foods may constitute a large part of the diet of urban birds [48]. For generalist and granivorous species, food availability is often high in urban environments due to the abundance of litter and other human-created food [49]. Prey availability for insectivorous and carnivorous species may also differ between urban and natural habitats. Despite its abundance, urban food is often of low quality in terms of macro- and micronutrients such as flavonoids and fatty acid content [50,51]. It is also well known that high calorie intake increases oxidative stress [52,53].

Birds mainly accumulate polyunsaturated fatty acids, which are particularly susceptible to free radical damage [54,55]. Omega-3 and omega-6 polyunsaturated fatty acids (PUFAs) are essential nutrients that birds must obtain from their diet as they cannot synthesize them themselves. For omnivorous bird species, the most important food sources include small fish, aquatic insects, and seeds such as sunflower and safflower. These fatty acids can be biochemically converted into long-chain, highly unsaturated forms that play an important role in various physiological functions [56]. Their importance is particularly evident in urban environments, where they help maintain the integrity of cell membranes, support heart and brain function, enable reproduction, promote feather health, and boost immune defenses [56]. High levels of fatty acids in the diet are associated with inflammation, which in turn is associated with oxidative stress.

A second group of stressors in wild birds includes various stressors such as elevated temperature or humidity, chemical pollution, radiation exposure, captivity, transportation, noise, and intense metabolic activity, such as migratory flight, which puts their bodies in a state of oxidative stress [55]. It is progressively becoming clear that global warming is a current and growing threat to animal biodiversity and a major economic challenge [57,58,59]. In both vertebrates and invertebrates, climate change can directly alter the oxidative status and fecundity rate of animals through heat stress or indirectly through scarcity of water and food, ocean acidification, or increased toxicity of pollutants [58]. Migratory flights pose many physiological challenges, such as energy production and maintenance of body homeostasis [55], to which many specific adaptations have evolved. Studies of the oxidative status of birds during their migratory phase or in long-distance flight have shown that long-distance flight is an additional source of oxidative stress. Migration is very stressful for the avian body and it has been found that metabolism and fasting are increased during prolonged travel [55]. This high metabolic activity associated with long-distance flights can reduce mass and fat stores [60], increase hematocrit [61], and increase the production of ROS, consequently damaging cell membranes, proteins, and DNA, leading to disease and cell death [15]. Most studies on the development of oxidative stress during the migration phase have been conducted on homing pigeons (*Columba livia domestica*), which are strong flyers but not true migrants. It was found that the redox status of these animals shifted towards oxidative stress after a 5.2 h flight compared with a 1.3 h flight or no flight at all [62]. It has also been found that intense physical activity can lead to inflammation and activation of immune cells, which further increases oxidative stress [63]. Predation risk can have significant effects on animal behavior and population dynamics [64,65,66]. This is known to trigger a physiological stress response, which may ultimately affect the birds’ body condition, glucocorticoid production, and cellular stress protein levels [67,68]. Recent research suggests that the risk of being preyed upon by wild birds may also contribute to oxidative stress. Studies conducted in various taxa in captivity have shown that animals exposed to predator stimuli exhibit altered antioxidant enzyme activities, increased oxidative stress and damage, increased metabolic rates, and altered corticosterone concentrations [47]. Urban environments are rich in novel resources for wildlife but also contain many potential sources of pro-oxidants. The effects of pollutants on redox balance are well known and have been used by ecotoxicologists for decades, with various tests to determine biomarkers of pollution or poor health [69]. Air pollution has been linked to many chronic diseases in humans, including asthma, cardiovascular disease, and cancer, often requiring hospitalization and leading to increased mortalitity rates [70,71]. Much less is currently known about the health of wild birds. Anthropogenic environmental pollutants that correlate with the occurrence of these diseases include ozone, sulfur dioxide, organic pollutants, heavy metals, nitrogen oxides, carbon monoxide, sulfur oxides, etc. These compounds act as pro-oxidants at the cellular level and pose a real health problem for humans and wildlife [72,73,74]. Birds, especially birds of prey, are particularly vulnerable due to their tropical habitats, long lifespan, and extensive habitats, resulting in high concentrations of pollutants [75,76]. Elevated concentrations of organic pollutants in birds of prey have been associated with physiological, neurological, and/or reproductive dysfunction [77], and recent studies have also reported the induction of oxidative stress responses [78,79]. According to the meta-analysis on polluted areas, including both cities and industrial areas, animals living in these areas experienced overall increased oxidative stress as well as increased levels of enzymatic antioxidants [74]. Both heavy metal exposure and exposure to car exhaust have been associated with negative oxidative responses in passerine birds [80,81]. In birds of prey, oxidative stress induced by heavy metal poisoning has been well studied, and thresholds have been proposed [82,83]. Heavy metals such as lead, cadmium, and mercury stimulate the overproduction of ROS, which disrupts antioxidant defenses and causes oxidative damage to lipids, proteins, and DNA, eventually culminating in apoptosis and necrosis of cells [84]. Among heavy metals, lead is considered a persistent global threat due to the millions of tons of lead mined historically and in recent years [85]. The sources of lead exposure for wildlife are not only related to mining, but also to urbanization, hunting, and fishing [85]. Birds may be particularly susceptible to air pollution because they have a unique respiratory system with air-flowing alveoli that is more than twice as efficient at gas exchange as the respiratory system of mammals [86]. Due to their lipophilic nature, organic pollutants preferentially accumulate in lipid-rich tissues such as fat, muscle, liver, and kidney, rather than in lipid-poor tissues such as feathers and blood [87,88].

Some studies have also reported negative effects of noise on oxidative balance in birds [89,90], while others found no significant effects [91,92,93,94]. Most of these studies focused on nesting rather than adult birds. Although the underlying physiological responses to short-term, controlled exposure to specific pollutants are relatively well understood, responses to and the consequences of long-term exposure to a variety of pollutants, e.g., in urban habitats, are less well understood but are thought to have more adverse health effects [70,95,96].

A third group of stressors includes various bacterial, fungal, or viral diseases and allergies. All these conditions stimulate the formation of free radicals by reducing the coupling of oxidation and phosphorylation in the mitochondria, resulting in increased electron loss and overproduction of free radicals [47]. In general, studies report a higher abundance of bacteria, viruses, and internal parasites in urban birds compared with non-urban populations [97,98]. At the molecular level, some clinical studies suggest that oxidative stress may be the physiological mechanism that promotes viral activation [99,100,101]. In cases of viral disease, oxidative stress may make cells more susceptible to viral activation and replication. This possible link between oxidative stress and viral activation is also supported by several studies that have found that antioxidant administration can reduce oxidative damage and viral load [102,103]. A recent meta-analysis across many different domestic species supported the hypothesis that the molecular mehanism of oxidative stress is likely to be responsible for the pathologic effects of herpesvirus infection [104]. The meta-analysis showed that herpesvirus decreases non-enzymatic antioxidant levels, increases the generation of reactive oxygen species, and causes oxidative damage to biomolecules [104].

### 3.1. The Relationship Between Hormones, Glucose and Oxidative Damage in Wild Birds

Wild birds have various adaptation mechanisms to current and changing environmental conditions. Physiological traits are key to these flexible adaptive measures that enable ready response to changes in extrinsic and intrinsic conditions [106].

Hormones play an important regulatory role in animals’ physiological networks as they can influence several physiological subnetworks such as energy metabolism, oxidative balance, and the immune system through their pleiotropic effects [106]. The crucial hormone for regulating glucose metabolism and life history decisions in birds is corticosterone [107,108]. Corticosterone is produced by the hypothalamic–pituitary–adrenal axis and regulates a wide range of physiological and behavioral traits both under normal conditions and in response to various stressful stimuli [108,109]. The baseline concentration of corticosterone maintains the energy balance required for predictable daily and seasonal rhythms [108]. A moderate increase in baseline corticosterone concentrations can improve innate immune system activity or reduce oxidative stress [110]. However, high corticosterone concentrations caused by acute stress can also cause oxidative stress, even after short-term exposure to acute stressors [111,112,113].

The blackbird (*Turdus merula*) is among the most prevalent songbird species in European cities [114]. Hand-reared urban blackbirds showed an attenuated hormonal stress response following exposure to a stressor compared with rural blackbirds [115], suggesting that they may be less vulnerable to stressors in terms of their endocrine characteristics. Corticosterone concentrations were also shown to be very high in birds without visible fat stores and with emaciated breast muscles [116]. These results led to the assumption that migratory birds with good fat and protein stores cope better with endurance flights and therefore do not perceive the phase of fasting and simultaneous high exertion as stressful [116].

The exact understanding of how acute stress affects oxidative damage is still scarce.Another feature that is subject to tight homeostatic control is glucose. Glucose is an important source of energy in many organisms, but it is also a putative trigger of oxidative stress that impairs cellular function [117]. Several neuroendocrine hormones regulate glucose concentration and can have both direct and indirect effects on oxidative balance, affecting cellular integrity [118]. Birds are among the most compelling candidates for studying these relationships because, despite their high blood glucose concentrations, birds have lower oxidative stress levels and live longer than mammals of comparable size [62]. This suggests that birds probably have physiological adaptations to circumvent the deleterious effects of hyperglycemia [117].

### 3.2. Effects of Reproduction and Immune Response on Oxidative Stress in Wild Birds

Currently, very little is known about the physiological mechanisms underlying the costs of reproduction in birds [114]. Reproduction is a critical and demanding phase in the life of animals, and in wild birds, reproduction has been reported as one of the cost factors associated with oxidative stress [62,119,120,121]. Reproduction may not be very costly if environmental conditions are favorable. Variations in the quality of the habitat in which an animal lives can have a significant impact on its reproductive activities [114]. In a poor-quality habitat with low food availability, breeding individuals are faced with metabolically intensive foraging and nutrient deficiency, which is likely to lead to increased generation of oxidative stress [122,123] and induction of pro-inflammatory proteins (e.g., haptoglobin) that limit the spread of oxidative damage in various tissues [124]. On the other hand, reproduction under good environmental conditions may attenuate the intensity of such trade-offs [123]. A consequence of this could be that reproductive activity leads to stimulation of physiological self-preservation mechanisms when it represents low stress for the organism. This would explain why several experimental reports found no change [114] or even a decrease in oxidative damage [125] or an increase in antioxidant protection [126] during reproduction. The rapid cell proliferation in the gonads and the allocation of antioxidants to the germ cells to improve their survival is often at the expense of the general antioxidant protection of the body [17,38]. In the male reproductive system, numerous factors are associated with oxidative stress on cellular and individual levels. At the cellular level, sperm metabolism and the occurrence of immature, abnormal, or dead spermatozoa are associated with overproduction of ROS, which can impair sperm function and oocyte maturation and reduce gamete quality over time [17,38]. In addition, activation of leukocytes following inflammation or infection has been shown to induce oxidative stress in spermatozoa. In general, the ability of males to cope with oxidative challenges varies greatly from individual to individual and is related to individual characteristics such as age, behavior, and social rank [127]. Females can further increase their oxidative stress levels during reproduction by providing an oxidative shield to their offspring, often sacrificing their antioxidant defences during non-reproductive periods [128]. Since gamete quality and body condition are directly related to reproductive potential, oxidative stress is closely linked to individual fitness. Oxidative stress in breeding wild birds can affect the laying date, which in turn can affect breeding success [129]. This is very important for long-distance migrants such as the barn swallow (*Hirundo rustica*), as only those birds that can tolerate the antioxidant costs of early arrival [130] are able to breed early and raise multiple broods per season [131]. The change in antioxidants and oxidative damage during the breeding season could be related to the change in food supply, as proteins and especially amino acids serve as precursors for some antioxidants [3,132]. A recent meta-analysis showed that oxidative damage in various tissues is higher in breeders with high reproductive effort than in breeders with low reproductive effort [128]. However, to date, there have been no comparative phylogenetic studies that measure oxidative damage and assess its relationship to reproductive effort, survival, and lifespan [62,133,134,135]. Calhoon et al. [136] compared slow-living tropical bird species with their fast-living temperate sister taxa and found that tropical species have longer life expectancy.

Oxidative balance is also closely linked to the immune response and inflammation in wild birds [50,137,138]. During the inflammatory response, inflammatory mediators such as cytokines and eicosanoids attract immune cells, including leukocytes and macrophages, and cause them to release ROS/RNS, in a process known as oxidative burst, to eliminate pathogens [8]. However, this can also cause collateral damage to cells, leading to immunopathology [3]. Inflammation-related proteins such as haptoglobin can limit the spread of oxidative damage in tissues by binding molecules via their pro-oxidant activity [124]. Chronic inflammation can increase oxidative damage and deplete antioxidants, potentially causing greater damage than the propagation of pathogens themselves [139]. Although this effect may be less pronounced in birds, tolerating pathogens may be less costly than initiating a protracted immune response [137,140].

According to a meta-analysis by Costantini [141] and an article by Costantini and Møller [137], the immune response in wild birds is significantly correlated with an increase in oxidative stress markers. In his meta-analysis, Costantini [141] compared young (taken before sexual maturity) and adult individuals, assuming that young animals suffer more from oxidative stress than adults because they have immature antioxidant mechanisms. The results showed that a change in oxidative status did not correlate with severe oxidative stress and that immune response did not lead to increased oxidative damage or decreased antioxidant levels. Sex-specific characteristics in birds, such as plumage coloration or the complexity of song, can indicate the oxidative status of an individual [142]. While carotenoids, which act as antioxidants in vitro, do not appear to have the same effect in vivo [137,143], melanin-based traits, which are also subject to sexual selection, are more frequently affected by oxidative stress [144]. In some cases, melanin coloration may even be indicative of cognitive abilities that, in rodents, have been shown to depend on oxidative stress [145]. However, studies linking birdsong to oxidative stress have produced mixed results [146]; so, this area of research remains unresolved.

Since oxidative stress is thought to affect almost all major life-history traits, it is considered an important mediator of life-history trade-offs [17]. Unfortunately, experimental results often do not provide clear evidence for this role. Oxidative stress is usually measured by changes in a few antioxidant or oxidative damage markers [62]. However, the redox status of an organism depends on a delicate balance between pro-oxidants and antioxidants, each of which has distinct regulatory functions [9]. An imbalance between the production of free radicals (ROS, RNS) and the antioxidant defence leads to increased concentrations of ROS or RNS and thus, to oxidative stress [4].

It is well established that gender and age can also influence the oxidative status of the individual. In zebra finches (*Taeniopygia guttata*), for example, males appear to be more susceptible than females to oxidative stress caused by increased breeding effort [147], which could be influenced by their early developmental conditions [148]. In contrast, in northern elephant seals (*Mirounga angustrirostris*), both sexes show increased oxidative stress during the breeding season, but differ in the type of oxidative damage: males show increased lipid and DNA damage, whereas females show increased protein damage [149].

### 3.3. Effects of Virus-Induced Oxidative Stress

Viral infections activate innate immune cells, triggering the release of ROS and pro-inflammatory cytokines. This activation also increases iron uptake by the mononuclear phagocyte system (also known as the reticuloendothelial system). Viruses further contribute to oxidative stress by promoting the formation of oxidants such as superoxide and nitric oxide (NO) while impairing the synthesis of important antioxidant enzymes such as catalase, superoxide dismutase, and glutathione peroxidase (GPx). The reduced presence and activity of these enzymes weakens immune function, as immune cells require a higher antioxidant capacity than most other cell types. In response to viral challenges, granulocytes and macrophages increase ROS production, which plays a critical antimicrobial role in defence against pathogens [150].

Avian influenza, which is caused by the avian influenza virus (AIV), is a particularly severe zoonosis. Infection with the AIV virus leads to a significant influx of inflammatory cells and stimulates NADPH oxidase activity, resulting in increased ROS levels [150]. This oxidative imbalance disrupts cellular redox homeostasis and triggers apoptosis in chicken oviduct epithelial cells via mitochondrial signalling pathways. Nitric oxide is associated with the disease process, and studies have shown that suppression of NO synthesis can improve survival from influenza. In addition, there is evidence that reactive species contribute to increased mortality, lung tissue damage, and inflammation during influenza infection in birds [150].

## 4. Biomarkers of Oxidative Stress in Wild Birds

Various environmental stressors such as pollutants, radiation, captivity, intense physical activity, diet, etc. influence oxidative stress in wild birds, and its measurement provides useful information on the health status of the organism. Determining the level of oxidative stress using biomarkers is a good indicator of living conditions in ecosystems [151]. Biomarkers are quantifiable indicators that allow quantification of a process. A good biomarker must have a measurable property that increases with increasing oxidative stress, must be stable and easily accessible, and should not change in the absence of oxidative stress [151]. Biomarkers can be measured in different samples, such as blood plasma, serum, red blood cells, or homogenates of different tissues, and their levels depend on the type of sample in which they are determined [12,152,153].

There are direct and indirect methods for the detection and quantification of free radicals and pro-oxidants. Direct methods measure the amount of ROS, which is very difficult because free radicals have a very short half-life [35,154,155]. Therefore, indirect methods are used that measure changes in the activity or amount of antioxidant components or that measure the amount of product resulting from damage to biomolecules [156]. A single measure of oxidative stress is insufficient to capture its complexity [12,157,158]. Different damaged biomolecules vary in their degradation and repair times [159], and tissues are differently susceptible to damage. Therefore, selection of the appropriate measurement methods and tissues is crucial. Furthermore, when selecting combinations of biomarkers, it is important to consider both pro-oxidant and antioxidant markers [3,160], as well as multiple biomarkers within each category. This ensures a comprehensive assessment of antioxidant defenses, oxidative damage, and pro-oxidant formation [161]. Unfortunately, ecological studies often require non-invasive methods that allow repeated sampling from small, easily accessible tissue samples [162]. Consequently, the measurement of redox status in wildlife relies on a limited number of biomarkers of oxidative stress that can be obtained from blood or other tissues that are easy to assess. These limitations complicate efforts to link oxidative stress to life course.

### 4.1. Biomarkers of Oxidative Damage in Wild Birds

Oxidative damage in wild birds can be detected by measuring lipid peroxidation, DNA damage, and protein carbonylation [159]. Membrane fatty acid composition is a critical factor influencing tissue susceptibility to ROS and may explain differences in lifespan between species [39,62].

Lipids are highly susceptible to oxidative damage through lipid peroxidation [62,163]. Accumulation of ROS can lead to lipid peroxidation and disrupt the arrangement of the lipid bilayer in the membrane, which can inactivate membrane-bound receptors and increase tissue permeability [164]. In addition to body condition, abiotic factors such as temperature are also associated with increased lipid peroxidation in light-bellied brent geese (*Branta bernicla*) [165]. Notably, body condition has no effect on lipid peroxidation in this species, suggesting that the relationship between redox homeostasis and body condition may be species-specific or at least tissue-specific [166]. In addition, migratory birds generally have higher lipid peroxidation levels and higher non-enzymatic antioxidant capacity than non-migratory birds, indicating a fundamental difference in redox balance due to migration and fasting [167]. In birds participating in long migrations, circulating antioxidant capacity increases while lipid peroxidation decreases during the pre-migratory period [164].

Lipid peroxidation products, including MDA (malondialdehyde) and unsaturated aldehydes, can inactivate various cellular proteins through the formation of cross-linkages. These products have also been used as indirect biomarkers of oxidative damage [164]. Mitochondrial DNA is particularly susceptible to oxidative damage by ROS because it is close to the site where mitochondrial ROS are produced and has limited protection and repair systems [168,169]. It has been suggested that mitochondrial dysfunction caused by oxidative DNA damage triggers an accelerated cycle of ROS production and thus, further damage [170]. Rapid growth can therefore lead to increased production of ROS with deleterious effects on mitochondrial DNA, limiting developmental growth.

Studies in vertebrates also show that fast-growing individuals exhibit higher oxidative stress and oxidative damage [171,172,173]. ROS can also induce DNA modifications via various other mechanisms, including base degradation, single- or double-strand breaks, or changes in purines, pyrimidines, or sugar bonds, as well as mutations, deletions, translocations, and protein cross-linking. Many of these DNA modifications play an important role in the development of cancer, aging, and neurodegenerative, cardiovascular, and autoimmune diseases. Among them, the formation of 8-OH-G (8-hydroxyguanosine) is the best-known indicator of oxidative stress-induced DNA damage and serves as a potential biomarker for carcinogenesis [164].

Proteins are the compounds most sensitive to oxidative damage, and they also scavenge a large proportion of ROS [174]. However, unlike lipids, oxidation of proteins is usually irreversible and dysfunctional oxidized proteins must be degraded by the proteasome [175]. The most commonly used method to determine oxidative damage to proteins is the measurement of protein carbonyls, concentrations of which increase under oxidative stress [159]. Protein carbonylation is the result of free radicals reacting with proteins and leading to the formation of a carbonyl group bound to the protein [176]. Most antioxidants neutralize ROS before they cause damage, which is more efficient in terms of energy than repairing damaged proteins [177]. The quantity of protein carbonyl groups provides a meaningful measure of oxidative damage, as carbonylation occurs very quickly after oxidative stress.

Despite reduced formation of ROS, high uric acid levels and the potential benefits of fasting, some birds exhibit oxidative stress due to biotic and abiotic factors associated with avian migration [178,179]. European robins captured during migration show increased protein carbonyl and GPx activity in red blood cells compared with resting birds, with both parameters correlating negatively with protein but not fat stores [180]. The carbonyl group is highly stable and can be detected by various tests, including Western blotting, enzyme-linked immunosorbent assay (ELISA), or spectrophotometric colorimetric assays [181].

### 4.2. Criteria for Biomarkers in Wild Birds

There are numerous excellent review articles on biomarkers to assess ROS, antioxidant defenses, oxidative damage, and associated repair mechanisms [3,182,183,184]. It is considered fundamental in research to demonstrate that changes in biomarkers reflect the subsequent development of disease [157]. The following conditions apply:

1. A biomarker should indicate most of the oxidative damage to the target molecule in vivo;
2. The selected biomarker should be stable and not be lost or artifactually formed in stored samples;
3. The biomarker must use a validated measurement technique. Validation criteria include intrinsic qualities such as specificity and sensitivity;
4. Sample collection should only minimally interfere with the normal life activities of the organism under investigation;
5. The biomarker must not be influenced by diet. This problem has already been noted with some of the strongest markers of oxidative damage, i.e., plasma MDA and HNE concentrations [182]. Another popular measure, total antioxidant capacity (TAC) of plasma, correlates very strongly with plasma uric acid concentrations, which may be an indicative of incidental amino acid deficiency rather than regulated antioxidant protection.

As Halliwell and Gutteridge [3] point out, no currently used biomarker of oxidative damage meets all of these technical criteria, but some are better than others.

## 5. Antioxidative Defence

The organism protects itself from the harmful effects of ROS through its antioxidant system. Antioxidants are substances that neutralize the effects of free radicals in small amounts and over a short period of time via three levels of protection: prevention, scavenging, and repair [10,185]. They are produced in the cell or ingested with food and act in different ways [186]. The components of the antioxidant system can be divided into enzymatic and non-enzymatic antioxidants [187]. As their activity generally increases during oxidative stress, they are the most important biomarkers of lipid, DNA, and protein damage, as their activity can be easily measured [156]. By measuring the activity of antioxidant enzymes, we can assess the oxidative status and the strength of the antioxidant response to oxidative stress [176].

The main enzymatic antioxidants involved in the neutralization of ROS in wild birds are superoxide dismutase (SOD), catalase (CAT), and glutathione peroxidase (GPx). Non-enzymatic antioxidants include metabolic and dietary antioxidants [10,34,188,189].

### 5.1. Enzymatic Antioxidants

#### 5.1.1. Superoxide Dismutase

Superoxide dismutase is the major enzymatic antioxidant that catalyzes the dismutation of the superoxide anion into molecular oxygen and hydrogen peroxide [12,190]. Superoxide dismutase can be considered both a pro-oxidant and an antioxidant enzyme, as it converts highly reactive superoxide radicals into the more stable hydrogen peroxide, a form that is more favorable for redox signaling purposes [191]. It is categorized as a metalloenzyme, which means that it uses metal ions as cofactors. In birds, it occurs in three forms that differ from each other based on the properties of the metal ion they contain, as well as amino acid composition, molecular mass, localization, regulatory mechanisms, and function. The first form or MnSOD (SOD2) is localized in the mitochondria, CuZnSOD (SOD1) is found in the cytosol, blood, lysosomes, and nucleus as well as between the inner and outer mitochondrial membranes, and SOD3 is specific to plasma [3,192].

In studies on wild white storks in Spain, Oropesa et al. [192] showed that SOD activity was more pronounced in adults than in juveniles, indicating that the superoxide radicals produced were effectively scavenged by the activity of this enzyme.

#### 5.1.2. Catalase

Catalase (CAT) is an enzyme that catalyzes the disproportionation of hydrogen peroxide into oxygen and water. It is most commonly found in peroxisomes, where the highest production of hydrogen peroxide occurs [3,193]. In addition to peroxisomes, catalase is also found in mitochondria, chloroplasts, in the cytosol, and outside the cell as a free or membrane-bound enzyme [194]. CAT can remove the hydrogen peroxide produced in the mitochondria only if the reactive oxygen species diffuses from the mitochondria into the peroxisomes [3]. However, once hydrogen peroxides enter the peroxisomes, they can be directly acted on by CAT and no cofactor is required to drive the reaction [39]. Reduced catalase activity has been described in various bird species at polluted sites compared with reference sites [195].

#### 5.1.3. Glutathione Peroxidase

Glutathione is another very important antioxidant that deserves our attention. Glutathione peroxidases are a group of enzymes that catalyze the reduction of hydrogen peroxide to water and oxygen at the expense of the oxidation of glutathione. In contrast to catalase, they can also reduce organic peroxides to alcohols and oxygen; this includes organic peroxides resulting from membrane damage. Several of the important GPXs that act as antioxidant enzymes are selenoproteins [196]. Four major selenium-dependent GPXs have been identified in mammals and birds in different tissues and subcellular locations [196,197]. GPx1 is found in red blood cells, liver, lung, and kidney and is restricted to the cytosol, nucleus, and mitochondria. GPx2 is found in the gastrointestinal tract and is restricted to the cytosol and nucleus of cells. GPx3 is found in plasma, kidney, lung, epididymis, vas deferens, placenta, seminal vesicles, heart, and muscle and is located in the cytosol or secreted into the plasma. GPx4, also known as phospholipid GPx, is widely distributed in all tissues and is found in the nucleus, cytosol, and mitochondria as well as in a membrane-bound form [198]. Research has shown that plasma glutathione may be a reliable marker of environmental stress in wild great tits (*Parus major*) [12,199]. Ingestion of some environmental substances, such as environmental toxins, can impair enzymatic antioxidant defenses. High activity of SOD, CAT and GPx was found in white stork chicks (*Ciconia ciconia*) from areas close to copper production facilities in Poland [200].

### 5.2. Non-Enzymatic Antioxidants

A smaller portion of antioxidant protection is provided by non-enzymatic antioxidants, proteins, and low-molecular compounds produced during metabolic processes. These include glutathione and lipoic acid, as well as uric acid, which is one of the most important antioxidants in birds. Glutathione is synthesized in cells from amino acid precursors and has, among many other important biological functions, an important role as a free radical scavenger. Although most previous studies have focused on the use of glutathione under laboratory conditions, Isaksson et al. [199] investigated the glutathione status of small songbirds (*Parus major*) in an urban ecological environment. Adult birds living closer to the city center had paler carotenoid coloration and higher levels of oxidized glutathione than those living more rurally, but the same pattern was not observed in nestlings [199]. In birds, uric acid is the major form of nitrogen excretion and an indicator of amino acid degradation, whereas in mammals it is the major end product of purine metabolism [201]. The antioxidant role of uric acid in both captive and wild birds has been hypothesized [201]. Uric acid varies within species, according to sex, weight, and heredity in different strains of domesticated turkeys, according to the degree of metabolic activity in pigeons, and according to stress in wild-caught house sparrows (*Passer domesticus*) and gray catbirds (*Dumetella carolinensis*) [202]. Cohen et al. [201] found that circulating uric acid was strongly correlated with circulating antioxidant levels in 526 individuals of 92 bird species, suggesting that part of the circulating antioxidant capacity may have been due to uric acid. Two new findings on uric acid in wild birds may expand our understanding of antioxidant physiology. First, allantoin is an oxidation product of uric acid, and the balance between these two molecules was found to be related to the exercise status of captive white-headed sparrows (*Zonotrichia leucophrys gambelii*), suggesting that this ratio may be an even better indicator of oxidative stress in birds than uric acid, as it is in humans [203].

Some of the non-enzymatic antioxidants are supplied to the body from the outside, for example, through food, i.e., vitamins E, C, A, and K, and minerals such as selenium and zinc [34,188,189]. Carotenoids and vitamin E, for example, may also play a role in neutralizing ROS, but their importance as functional antioxidants in wild birds is controversial [143]. According to a meta-analysis, researchers reported a weak positive correlation between dietary carotenoids and antioxidant capacity, but no correlation between dietary carotenoids and oxidative damage. More recent studies on the effects of carotenoids in tissues have reported both positive correlations with liver mitochondrial function and no correlation with tissue carotenoid levels or lipid peroxidation [204]. In an experiment on carotenoid supplementation in captive zebra finches (*Taeniopygia guttata*), which exhibit sexually selected red beak coloration, Alonso-Alvarez et al. [147] found that an increase in systemic carotenoid levels was positively associated with an increase in antioxidant defense. Since then, the antioxidant effect of carotenoids in birds has been the subject of intense debate [205,206]. Despite the numerous studies in this field demonstrating the benefits of carotenoids for growth, health, reproduction, and survival, we still know very little about the supply and requirements of carotenoids in wild birds.

Due to its molecular properties, vitamin E is one of the most effective free radical scavengers in animals [207]. It is mainly found in green leafy vegetables and has clear positive effects on the antioxidant capacity of the serum of young broiler chickens (*Gallus domesticus*) [201] and on the growth rate of nestlings of barn swallows (*Hirundo rustica*) [208] when consumed and accumulated in high amounts. However, more recent studies on the antioxidant effects of vitamin E in adult wild birds are less conclusive. A comparative study of nearly 100 New World bird species showed that vitamin E levels are poorly related to other circulating micromolecular antioxidants and to variations in life history (e.g., survival time, breeding time, nestling time, basal metabolic rate, body mass, etc.) [202,209]. Some researchers have speculated on the valuable lipid-protective role of vitamin E in birds, where flight and fatty acid metabolism are linked, but little evidence has been found [209].

## 6. Conclusions

Oxidative stress plays a crucial role in influencing the health and survival of wild birds. It occurs when the overproduction of reactive oxygen species in oxygen-consuming metabolic processes leads to an imbalance in which pro-oxidants are favored over antioxidants. ROS are generated by cellular metabolic activities and various environmental stressors during key stages such as migration and in response to diseases that affect birds in their natural habitat. Due to their unpaired electrons, ROS are highly reactive molecules that interact with cellular macromolecules such as nucleic acids, lipids, and proteins, thereby disrupting their functions. As a result of this high reactivity, ROS production is tightly regulated by the body’s antioxidant defense system, which includes both enzymatic and non-enzymatic components. Investigating the role of oxidative stress in the development of disease in wild birds requires the use of appropriate biomarkers. Studies on oxidative stress in birds have shown that the use of multiple biomarkers is the most effective way to gain insight into these systems. The use of biomarkers of oxidative stress should always be complemented with markers of oxidative damage. Therefore, understanding the balance between ROS production and antioxidant defenses in wild birds is essential for comprehending how these species cope with different environmental and physiological challenges.

## Data Availability

The authors will make the data available upon reasonable request.
